# Knowledge, Attitudes and Perceived Preparedness Regarding Cardiopulmonary Resuscitation and Automated External Defibrillator Use Among Health-Related University Students: A Cross-Sectional Study

**DOI:** 10.3390/healthcare14060730

**Published:** 2026-03-12

**Authors:** Caterina Mercuri, Giovanni Marasco, Alessandra De Pasquale, Dario Marasciulo, Silvio Simeone, Adele Sarcone

**Affiliations:** Clinical and Experimental Medicine Department, Magna Graecia University, 88100 Catanzaro, Italy; giovanni.marasco1@studenti.unicz.it (G.M.); alessandra.depasquale@studenti.unicz.it (A.D.P.); dario.marasciulo@studenti.unicz.it (D.M.); silvio.simeone@unicz.it (S.S.); adele.sarcone@studenti.unicz.it (A.S.)

**Keywords:** cardiopulmonary resuscitation, automated external defibrillator, BLS-D training, healthcare students, knowledge and attitudes, rhythm recognition, perceived preparedness, out-of-hospital cardiac arrest, Italy

## Abstract

**Background:** Early cardiopulmonary resuscitation (CPR) and timely use of automated external defibrillators (AEDs) are critical determinants of survival following out-of-hospital cardiac arrest (OHCA). University students enrolled in healthcare degree programs represent a strategic target population for the dissemination of basic life support and defibrillation (BLS-D) skills. However, evidence on their level of knowledge, attitudes, and perceived preparedness remains limited in Southern Italy. **Methods:** A cross-sectional observational study was conducted between mid-December 2025 and 15 January 2026 among undergraduate healthcare students at the Magna Graecia University of Catanzaro (Italy). Data were collected using a structured, self-administered questionnaire assessing socio-demographic characteristics, CPR/AED knowledge, attitudes, and perceived confidence. Composite knowledge scores were calculated and categorized as poor, sufficient, good, or excellent. Statistical analyses included chi-square tests, Cramér’s V, and Spearman’s rank correlation. **Results:** A total of 604 students were included (mean age 24.4 ± 6.7 years; 69.9% female), of whom 46.4% reported prior BLS-D training. Knowledge levels were heterogeneous: myocardial infarction was widely recognized as a cause of cardiac arrest (81.1%), whereas recognition of non-shockable rhythms, including asystole and pulseless electrical activity, remained low (<25%). Procedural knowledge, particularly regarding the chain of survival and chest compression rate, improved with academic year and prior BLS-D training. Conversely, ventilation skills and correct AED pad placement were consistently inadequate. Attitudes toward CPR were largely positive; however, perceived confidence in performing resuscitation was moderate to low, especially in complex scenarios. More than 80% of students expressed strong interest in further training and supported mandatory BLS-D education. **Conclusions:** Healthcare students demonstrated favorable attitudes toward CPR but insufficient and uneven knowledge, particularly in rhythm recognition, ventilation, and AED use. Academic progression and structured BLS-D training were associated with improved competencies, although critical gaps persisted. Integrating mandatory, hands-on BLS-D training with regular refresher sessions into healthcare curricula should enhance preparedness and potentially reduce OHCA-related mortality, especially in high-risk regions such as Calabria.

## 1. Introduction

Cardiopulmonary resuscitation (CPR) and the use of an automated external defibrillator (AED) are essential life-saving interventions in the treatment of out-of-hospital cardiac arrest (OHCA), one of the leading causes of death worldwide [1]. The incidence of sudden cardiac arrest is estimated at between 55 and 113 cases per 100,000 inhabitants/year in Europe and North America, representing a major public health problem [2]. In Italy, approximately 60,000 OHCAs occur annually, with survival to hospital discharge rarely exceeding 5–10%, despite advances in cardiological and resuscitation care [2,3].

Early and high-quality intervention is a key determinant of survival following cardiac arrest [4]. Patients who receive immediate CPR have a two to four fold higher probability of survival compared with those who receive no treatment, highlighting the critical role of timely intervention by witnesses [5]. Survival rates further increase when CPR is combined with early defibrillation, as time represents the main determinant of the effectiveness of resuscitation efforts [6].

High-quality CPR is the operational foundation of OHCA management and is based on performing chest compressions at the appropriate frequency and depth, correct chest decompression, minimal interruptions, and appropriate ventilations volumes [6]. In this context, the “Chain of Survival” represents the conceptual framework linking early recognition of cardiac arrest, prompt activation of emergency medical services, high-quality CPR, early defibrillation, and post-resuscitation care as interdependent steps essential to improving survival outcomes. This model, endorsed by the American Heart Association (AHA) and the European Resuscitation Council (ERC), underscores the systemic nature of effective resuscitation care [7,8].

The timeliness of intervention is the most important prognostic factor: for every minute of delay in defibrillation, survival probability decreases by 7–10%, becoming almost zero after about 10 min in the absence of CPR and defibrillation [9]. However, the average response time of emergency services in Italy is estimated to be between 8 and 12 min in urban areas and can exceed 15–20 min in rural areas, creating a critical time window in which the intervention of bystanders is crucial [10,11].

Numerous studies have shown that bystander-performed CPR can double or triple survival rates, while the use of an AED within 3–5 min of cardiac arrest can increase survival by up to 50–70% [12]. The combination of CPR and early defibrillation is therefore the intervention with the greatest impact on reducing mortality in OHCA [10,12,13].

Within this scenario, students enrolled in health-related degree programs, including medicine, nursing, and other healthcare-related academic courses, represent a strategic population for the dissemination of basic life support and defibrillation (BLS-D) skills. As future professionals operating within healthcare systems, they are expected to intervene in emergency situations in accordance with professional ethical codes and are required to possess basic resuscitation competencies, now considered core skills across healthcare disciplines [14]. Moreover, during their academic training, they are frequently exposed to high-acuity clinical environments and may be required to assist in real resuscitation events, underscoring the need for both theoretical knowledge and practical competence [14,15].

Students enrolled in health-related academic programs also act as skill multipliers within the community, transferring knowledge to peers, family members, and the general population, participating in educational initiatives, and serving as role models for safe behaviors. Their widespread presence in public and healthcare settings further positions them as potential first responders in cases of OHCA.

Despite the recognized importance of BLS-D training, international literature consistently reports heterogeneous and often insufficient levels of preparation among university students [16,17,18]. Only a proportion of students receive structured BLS-D training, and skills tend to decline rapidly without regular refreshers, with significant deterioration already after a few months [19,20,21,22]. A persistent gap between theoretical knowledge and perceived operational confidence has also been described, with barriers such as limited curricular integration, lack of refresher courses, and fear of making mistakes or legal consequences [23,24].

In this perspective, the Calabria region has characteristics that make the enhancement of resuscitation skills particularly urgent. Cardiovascular mortality rates exceed the national average, with a high prevalence of major risk factors such as hypertension, obesity, and diabetes, alongside a progressively aging population [25,26]. These epidemiological challenges are compounded by geographical and organizational factors, including extensive mountainous and rural areas, longer emergency medical service response times in inland regions, and a low density of publicly accessible AEDs compared with national averages [27,28].

Despite extensive international evidence supporting the importance of BLS-D training, structured data on knowledge, attitudes, and perceived preparedness regarding CPR and AED use among healthcare students in the Calabrian context remain limited. To the authors’ knowledge, this is one of the first studies to comprehensively assess these dimensions in a Calabrian university setting.

Accordingly, this study aims to evaluate the level of knowledge, attitudes, and perceived preparedness of students at a Calabrian university with respect to cardiopulmonary resuscitation and the use of automated external defibrillators, through the administration of a structured questionnaire. The findings are intended to identify potential gaps in training and inform targeted educational interventions, ultimately contributing to improved preparedness of future healthcare professionals and a potential reduction in cardiac arrest-related mortality.

## 2. Materials and Methods

### 2.1. Study Design and Setting

A cross-sectional observational study was conducted to assess the level of knowledge, attitudes, and perceived confidence regarding CPR and the use of AED, among university students. The study was carried out at the Magna Graecia University of Catanzaro, Italy.

### 2.2. Study Population

The study population consisted of university students aged ≥18 years enrolled in undergraduate healthcare degree courses, including Medicine and Surgery, Nursing, and other healthcare-related programs. Students were eligible regardless of previous attendance at BLS or BLS-D training courses.

Data collection was conducted between mid-December 2025 and 15 January 2026, during scheduled teaching activities involving students.

### 2.3. Inclusion and Exclusion Criteria

Inclusion criteria:

- Age ≥ 18 years;
- Enrollment in a healthcare-related undergraduate degree program;
- Voluntary completion of the questionnaire.

Exclusion criteria:

- Age < 18 years;
- Enrollment in non-healthcare degree programs;
- Incomplete or invalid questionnaires;
- Refusal to provide informed consent.

### 2.4. Sample and Recruitment

A total of 606 students completed the questionnaire. Participants were recruited through a convenience sampling strategy using an electronic questionnaire developed with Google Forms. The questionnaire was administered on a voluntary and anonymous basis exclusively during face-to-face teaching activities. The assessment of knowledge was not part of a BLS-D training session but was administered independently, although some participants had received prior BLS-D training. Approximately 1200 students were enrolled in the healthcare degree programs involved in the study during the academic year 2025–2026. Considering the 604 questionnaires included in the analysis, the estimated response rate was approximately 50.3%.

### 2.5. Data Collection Tool

Data were collected using a structured, self-administered questionnaire developed ad hoc by the authors based on the available scientific literature and the main international guidelines on cardiopulmonary resuscitation and BLS-D.

The questionnaire was reviewed by experts in the field to assess its clarity, relevance, and consistency of content and was preliminarily tested on a pilot group of students to verify its comprehensibility and feasibility. The internal consistency of the questionnaire was assessed using Cronbach’s alpha. The overall Cronbach’s alpha coefficient was 0.711, indicating good internal consistency.

The questionnaire was divided into three main sections, aimed at exploring the students’ socio-demographic characteristics, knowledge of CPR/AED, and attitudes.

Section A—Socio-demographic characteristics.

This section collected information on age, gender, marital status, province of residence, university, course of study, year of study, participation in BLS/BLS-D training courses, presence of healthcare professionals among family members, and family history of cardiovascular diseases.

Section B—Knowledge of CPR and AED.

This section assessed theoretical and practical knowledge regarding:

- Causes of cardiac arrest;
- Identification of shockable and non-shockable cardiac rhythms;
- Sequence of CPR maneuvers in adults;
- Use of a semi-automatic external defibrillator (AED).

Questions were formatted as multiple-choice or dichotomous (Yes/No/I don’t know).

Rhythm-recognition items were text-based and consisted of brief clinical descriptions specifying the cardiac rhythm; no ECG tracings or image-based rhythm strips were included.

Section C—Attitudes and perceived confidence.

This section explored:

- Ethical perception of resuscitation;
- Perceived confidence in performing CPR and using an AED;
- Willingness to intervene in the event of cardiac arrest;
- Perceived barriers to performing the maneuvers.

Responses were recorded using 5-point Likert-type scales or dichotomous options.

### 2.6. Study Variables

Independent variables included socio-demographic characteristics (age, gender, marital status), academic variables (degree program and year of study), previous BLS/BLS-D training, and family health background. Dependent variables included level of CPR/AED knowledge, composite knowledge scores, attitudes toward CPR, perceived confidence, and willingness to intervene.

### 2.7. Construction of Knowledge Scores

Based on the responses provided, composite knowledge scores were constructed relating to:

- Causes of cardiac arrest;
- Recognition of defibrillable rhythms;
- CPR procedures and AED use;
- Total knowledge score.

Each correct answer was assigned 1 point, while incorrect answers or “I don’t know” answers were coded as 0 points.

The total knowledge score could range from 0 to 27 points and was subsequently also expressed as a percentage.

The overall knowledge level was categorized as:

- Poor (<50%);
- Sufficient (50–69%);
- Good (70–89%);
- Excellent (≥90%).

These thresholds were applied following a conventional approach commonly used in Knowledge–Attitude–Practice (KAP) surveys, where percentage-based cut-offs are adopted in the absence of validated scoring standards. The categorization was used exclusively for descriptive purposes [29,30].

These cut-off values were applied exclusively for descriptive purposes.

### 2.8. Statistical Analysis

Statistical analyses were performed using Stata Statistical Software: Release 19.

Categorical variables were summarized as frequencies and percentages. Group comparisons were performed using the chi-square test (χ^2^) for categorical variables, with effect size estimated by Cramér’s V (thresholds: small ≈ 0.10; medium ≈ 0.30; large ≈ 0.50).

For analyses by year of course (1–6), in addition to χ^2^ tests on r × c tables, ordinal trend was assessed using Spearman’s rank correlation coefficient (ρ) where applicable. Statistical significance was defined as *p* < 0.05 (α = 0.05).

### 2.9. Ethical Considerations

Participation in the study was voluntary and anonymous. Before completing the questionnaire, all participants received information about the purpose of the study and the processing of data. Completion of the questionnaire was considered an expression of informed consent.

The study was conducted in accordance with the ethical principles of the Declaration of Helsinki and was submitted for evaluation to the competent Ethics Committee, which approved its conduct (protocol no. 303/2025).

The data were analyzed exclusively in aggregate form, in compliance with current legislation on the protection of personal data (EU Regulation 2016/679).

## 3. Results

The results are organized into sections describing the socio-demographic characteristics of the participants, prior exposure to BLS-D training, levels of knowledge regarding CPR and AED use, attitudes toward resuscitation, and perceived confidence, with comparisons across years of study and training status.

### 3.1. Socio-Demographic Characteristics of the Sample

The study involved a total of 604 university students. Participants’ age ranged from 18 to 50 years, with a mean age of 24.4 ± 6.7 years and a median of 22 years. More than half of the sample (54.5%) was between 18 and 22 years old, indicating a predominantly young population.

Gender distribution showed a clear prevalence of females (422 students; 69.9%), followed by males (180 students; 29.8%); two participants (0.3%) identified as non-binary.

Most students were single (86.9%), while 8.3% were married and 3.8% were cohabiting. Regarding religious affiliation, 80.5% identified as Catholic Christians, followed by atheists (10.6%) and agnostics (4.5%).

In terms of residence, almost half of the sample came from the province of Catanzaro (49.2%), followed by Cosenza (25.0%), Crotone (11.3%), Reggio Calabria (9.1%), and Vibo Valentia (5.5%).

The most represented degree course was Nursing (58.0%), followed by Medicine and Surgery (30.5%); the remaining students were enrolled in other courses in the health sector, each with percentages below 5%. In terms of year of study, 72.9% of students were enrolled in the first three years of their degree programs, while 27.1% were attending the fourth to sixth years.

**Figure 1:**
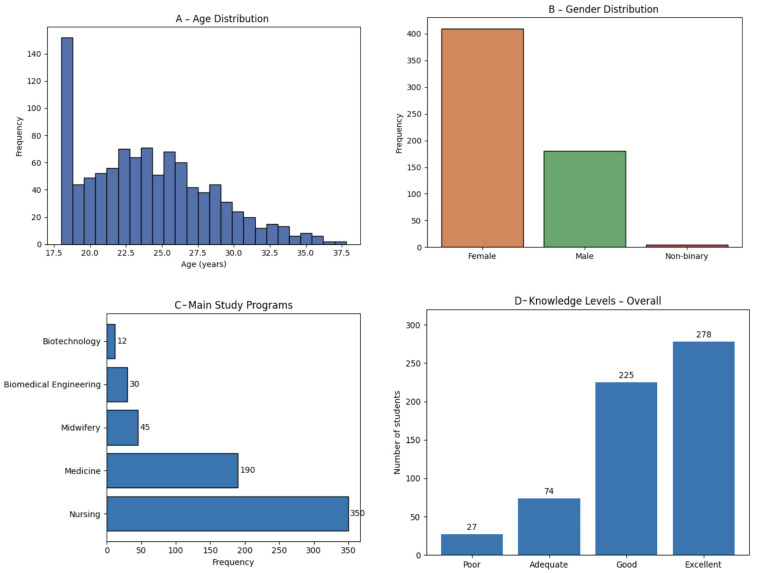
Socio-demographic characteristics of the study population. Panel (**A**) shows the age dis-tribution of participants, including mean and median values. Panel (**B**) illustrates gender distribu-tion. Panel (**C**) reports the main study programs attended by the students. Panel (**D**) presents the distribution according to year of study.

The socio-demographic characteristics of the study population are summarized in Figure 1.

### 3.2. Previous Training in BLS-D and Family Background

**Figure 2:**
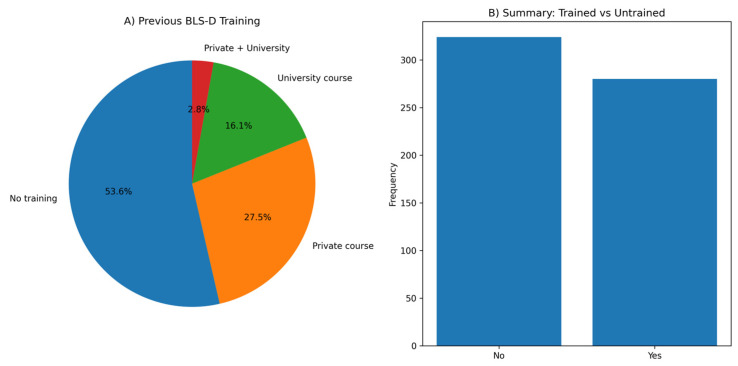
Previous BLS-D training among participants. Panel (**A**) displays the type of BLS-D training attended (none, private, university-based, or both). Panel (**B**) summarizes the distribution of trained versus untrained students.

Overall, 46.4% of students reported having attended at least one BLS or BLS-D training course. Specifically, 27.5% had attended a private course, 16.1% a university-based course, and 2.8% both. The remaining 53.6% of the sample had never received formal BLS-D training (Figure 2).

When asked about the availability of CPR/BLS-D training at their university, only 35.3% of students reported the presence of structured training, whereas 54.0% stated they were unaware of such educational opportunities.

In addition, 35.4% of participants reported having at least one family member working in the healthcare sector, and 35.8% reported the presence of cardiovascular disease among family members. Previous exposure to BLS-D training among participants is shown in Figure 2.

### 3.3. Knowledge of the Causes of Cardiac Arrest

The assessment of knowledge about the causes of cardiac arrest showed good overall awareness, although with a high degree of uncertainty for some conditions. Myocardial infarction was correctly recognized as a cause of cardiac arrest by 81.1% of students (sum of ‘agree’ and ‘strongly agree’ responses). Similar percentages were observed for ventricular fibrillation (60.1%) and ventricular tachycardia (47.0%).

Analysis by year of study showed a progressive improvement in theoretical knowledge: recognition of myocardial infarction increased from 63.6% in the first year to 93.8% in the sixth year; ventricular fibrillation from 45.5% to 93.8%; and ventricular tachycardia from 36.4% to 81.3%.

Conversely, for conditions such as atrial fibrillation, head trauma, and cardiac tamponade, a significant proportion of students reported uncertainty, with percentages of “unsure” responses exceeding 30%, indicating less consolidated knowledge.

### 3.4. Recognition of Heart Rhythms

With regard to the recognition of heart rhythms, 62.3% of students correctly identified ventricular fibrillation as a defibrillable rhythm, while only 48.2% correctly recognized pulseless ventricular tachycardia. The percentages were lower for non-shockable rhythms: asystole was correctly recognized by 24.7% of students and pulseless electrical activity (PEA) by 24.5%. In all these categories, a significant proportion of students selected the “I don’t know” response, indicating uncertainty.

A clear learning gradient emerged across academic years. Correct recognition of ventricular fibrillation increased from 50.0% in the early years to 100% among sixth-year students, while correct identification of pulseless ventricular tachycardia increased from 31.8% to 68.8%. Conversely, recognition of non-shockable rhythms remained consistently low and showed limited improvement with academic progression (asystole: 9.1% to 37.5%; PEA: 13.6% to 37.5%).

Inferential analysis confirmed these trends. The association between academic year and correct recognition of ventricular fibrillation was statistically significant (χ^2^ test, *p* < 0.001; Cramér’s V = 0.42, large effect). For pulseless ventricular tachycardia, the association was also significant (*p* = 0.002; Cramér’s V = 0.28, medium effect). For non-shockable rhythms (asystole and PEA), associations were weaker and not statistically significant (*p* > 0.05). Spearman’s rank correlation indicated a strong positive trend for ventricular fibrillation (ρ = 0.68, *p* < 0.001) and a moderate trend for pulseless ventricular tachycardia (ρ = 0.45, *p* = 0.004), while trends for asystole and PEA were negligible. Across all academic years, students who had attended BLS-D training demonstrated higher accuracy in identifying defibrillable rhythms compared with students without prior BLS-D training. This accuracy refers to the correct classification of text-based rhythm descriptions, as no ECG tracings or image-based rhythm strips were used. However, for non-shockable rhythms, performance remained low regardless of training status.

### 3.5. Knowledge of CPR Procedures and AED Use

Most students (69.5%) correctly identified calling the Single Emergency Number (112) as the first action to take when encountering an unresponsive person. Knowledge of the chain of survival was correct in 73.3% of cases. Regarding CPR procedures, 51.3% of students correctly indicated that ventilation should not be initiated first in adult CPR, while only 36.1% correctly identified the recommended compression rate of at least 100 compressions per minute. Correct hand placement was recognized by 67.2% of participants.

Procedural knowledge improved progressively with academic year. Knowledge of the chain of survival increased from 63.3% in first-year students to 93.8% in sixth-year students, while correct identification of compression frequency rose from 27.3% to 68.8%.

**Figure 3:**
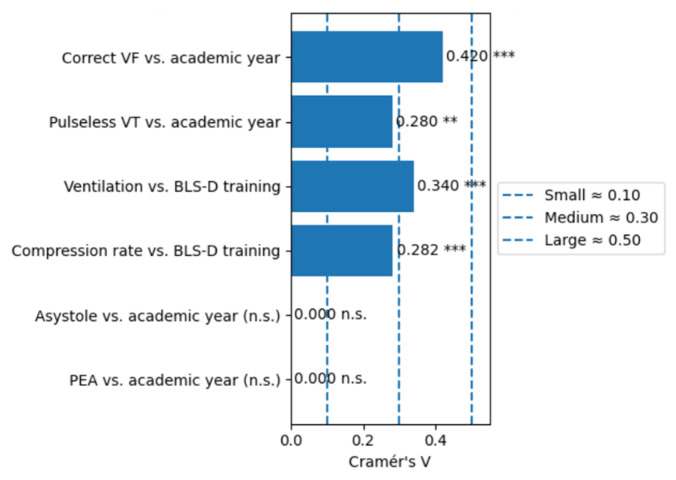
Effect sizes (Cramer’s V) for key associations. (*** *p* < 0.001; ** *p* < 0.01; n.s. = not significant).

**Figure 4:**
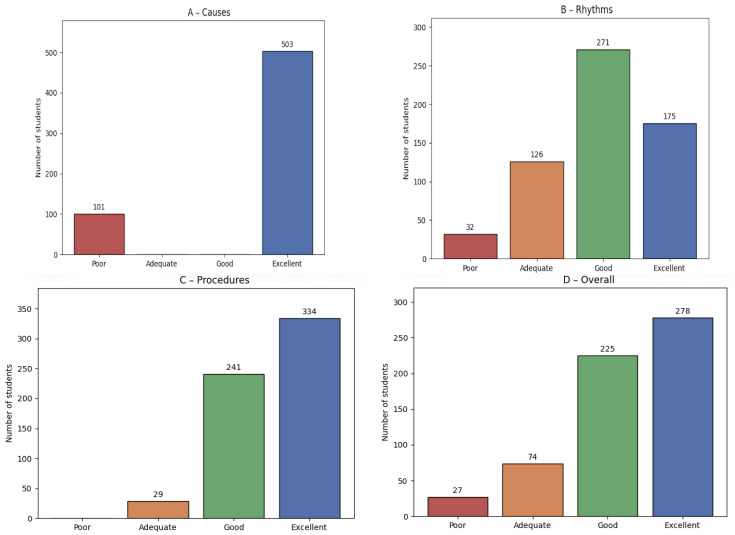
Categorization of knowledge levels (poor, adequate, good, excellent) for causes of car-diac arrest (**A**), rhythm recognition (**B**), CPR procedures (**C**), and overall knowledge (**D**).

Students who had attended BLS-D courses consistently demonstrated higher rates of correct responses, particularly regarding chest compressions and the chain of survival. In contrast, knowledge related to ventilation remained persistently low (<25%) across all academic years. Although BLS-D training was significantly associated with higher rates of correct responses for ventilation items, the absolute performance remained limited, indicating that training improved results statistically but not to a satisfactory level. With regard to AED use, only 43.7% of students correctly identified pad placement, whereas 78.6% correctly recognized that CPR can be initiated even in the absence of an AED. Considerable uncertainty persisted regarding technical aspects of AED use. Inferential analysis confirmed these patterns. For ventilation, the association between BLS-D training and correct responses was statistically significant (χ^2^(6) = 139.60, *p* < 0.001; Cramér’s V = 0.340), indicating a medium effect size, despite overall low performance. For compression rate, the association was also significant (χ^2^(6) = 96.39, *p* < 0.001; V = 0.282), suggesting a small-to-medium effect (Figure 3). Regarding academic progression, Spearman’s rank correlation revealed strong positive trends for compression frequency (ρ = 0.943, *p* = 0.0048) and chain of survival knowledge (ρ = 0.829, *p* = 0.0416), while ventilation responses also improved significantly across years (χ^2^ test, *p* < 0.001), although remaining at suboptimal levels. This confirms that both training and academic progression contribute to incremental improvements, but ventilation remains a persistent area of weakness. Figure 4 shows the categorization of knowledge levels (poor, adequate, good, excellent) for causes of cardiac arrest, rhythm recognition, CPR procedures, and overall knowledge.

### 3.6. Attitudes and Perceptions Towards CPR

The vast majority of students rejected the notion that CPR is ethically inappropriate (89.6%) or harmful to patients (82.5%) (Figure 4). Additionally, 62.6% believed that adequate training of future healthcare professionals can positively influence patient outcomes after cardiac arrest.

Although ethical attitudes were largely favorable, perceived confidence was moderate to low. Only 36.8% of students reported feeling very or fairly confident performing CPR on an adult, with confidence further decreasing in scenarios involving elderly or traumatized victims.

**Figure 5:**
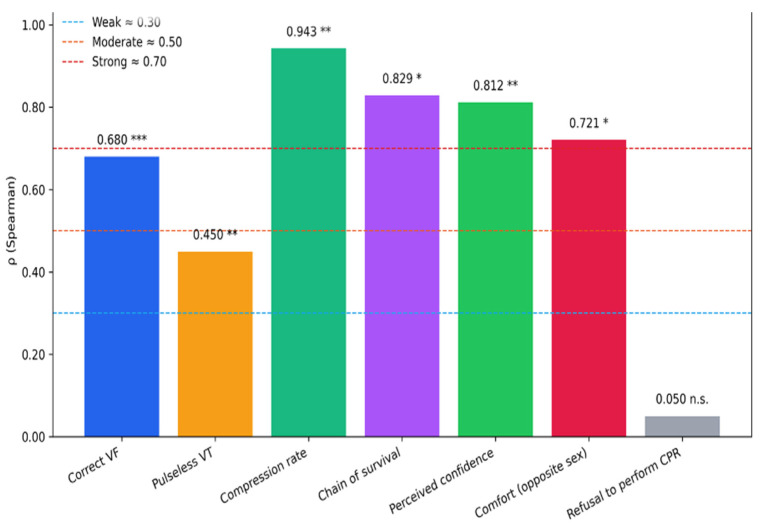
Spearman correlations (p) with academic year. (* *p* < 0.05; ** *p* < 0.01; *** *p* < 0.001; n.s. = not significant).

Analysis by year of study showed a progressive increase in perceived confidence, with the percentage of students who were very or fairly confident rising from 18.2% in the first year to 56.3% in the sixth year, especially in more complex scenarios such as trauma or intervention on strangers. Inferential analysis confirmed this trend as statistically significant (Spearman’s ρ = 0.812, *p* = 0.009), indicating a strong positive correlation between academic progression and confidence (Figure 5).

With regard to performing CPR on a person of the opposite sex, the percentage of students who said they were very or fairly comfortable increased from 68.2% in the first year to 81.3% in the sixth year, showing a positive gradient as the training progressed. This association was significant (Spearman’s ρ = 0.721, *p* = 0.018), while stratified analysis for BLS-D training showed comparable percentages between trained and untrained students (79.1% vs. 76.8%, χ^2^ test *p* > 0.05), suggesting that the sex of the victim does not represent a significant barrier to performing CPR, regardless of specific training.

Conversely, with regard to the possibility of refusing to perform CPR in cases of perceived inadequacy, the percentages of correct answers were low and essentially unchanged between the different years of the course, ranging from 54.5% in the first year to 50.0% in the sixth year. Chi-square analysis confirmed the absence of a significant association (*p* > 0.05), and Spearman’s correlation indicated no meaningful trend (ρ ≈ 0.05, *p* = 0.78). Similarly, stratified analysis based on attendance of a BLS-D course showed no significant differences between trained and untrained students (52.1% vs. 49.6%), indicating persistent decision-making and medico-legal uncertainty across the training program.

Finally, interest in learning more about CPR and AEDs was very high (84.6%), as was agreement on the need to enhance university training (86.1%) and make BLS training mandatory for both students and healthcare professionals (>83%).

Students’ attitudes, perceived confidence, willingness to intervene, and perceived barriers toward CPR are illustrated in Table 1.

## 4. Discussion

This study describes the knowledge, attitudes, and perceived confidence related to CPR and the use of AED in a large sample of university students enrolled in health-related degree programs. Overall, a “mixed” picture emerges: on the one hand, generally positive attitudes and a progressive improvement in several competencies as students advance through their academic years; on the other hand, persistent gaps remain in key areas, particularly the recognition of non-shockable rhythms, ventilation skills, decision-making and medico-legal aspects, and technical competencies related to AED use.

Knowledge of the causes of cardiac arrest appears moderate, with most students correctly identifying myocardial infarction as a primary cause. However, uncertainty emerged for less common conditions such as atrial fibrillation, cardiac tamponade, or head trauma, consistent with previous findings showing that students recognize common causes but struggle with more complex scenarios [17,31].

The recognition of defibrillable versus non-shockable rhythms revealed a critical area of concern. While approximately two-thirds of students correctly identified ventricular fibrillation as a defibrillable rhythm, only a minority could recognize asystole and pulseless electrical activity (PEA). This pattern is particularly troubling given that rhythm recognition is fundamental to appropriate defibrillation decisions during cardiac arrest management. Studies from other medical schools have reported similar deficiencies, with rhythm interpretation consistently identified as one of the most challenging aspects of resuscitation training [32].

A clear learning gradient emerged across academic years for several knowledge domains, confirming that curricular exposure contributes to improved theoretical understanding. However, some competencies, particularly the recognition of non-shockable rhythms, showed limited improvement, indicating that passive exposure alone is insufficient [33,34].

However, the persistence of poor knowledge regarding non-defibrillable rhythms across all academic years, even among senior students, indicates that passive curriculum exposure alone is insufficient. The limited improvement in recognition of asystole and PEA despite six years of medical education underscores the necessity for dedicated, hands-on BLS-D training that emphasizes rhythm interpretation through simulation-based scenarios [35,36].

Students with prior BLS-D training performed better in several domains, supporting evidence that structured, hands-on programs are more effective than theoretical instruction alone. Persistent deficits in ventilation skills, compression rate, and AED pad placement highlight areas requiring targeted reinforcement [36,37].

Knowledge related to ventilation remained low across all academic years and did not substantially improve after BLS-D training. This suggests that current programs may require revision to better emphasize this component. Similarly, fewer than half of students correctly identified AED pad placement, despite its central role in the chain of survival [38,39]. Given the low density of publicly accessible AEDs in rural and inland regions of Calabria, healthcare students represent a vital resource for potential bystander intervention when encountering cardiac arrest in community settings. Their inability to correctly operate an AED could represent a missed opportunity to improve survival outcomes in a region with elevated cardiovascular mortality.

The findings of this study must be interpreted within the specific epidemiological and organizational context of the Calabria region. As documented in recent reports from the Italian National Institute of Health, the South of Italy, including Calabria, continues to experience higher rates of cardiovascular mortality and hospitalizations for acute myocardial infarction compared to Northern and Central regions [40]. Calabria registers among the highest rates of years of life lost due to cardiovascular diseases among Italian regions [41,42].

These epidemiological challenges are compounded by geographical and organizational factors that limit timely access to advanced cardiac care. Rural and mountainous areas, longer Emergency Medical Services response times, and the low availability of public-access AEDs further reduce survival after out-of-hospital cardiac arrest [43,44,45].

In this context, the inadequate CPR and AED knowledge among healthcare students represents not merely an educational shortcoming but a significant public health concern. Future physicians, nurses, and other healthcare professionals educated in Calabria will serve communities where bystander CPR and early defibrillation may represent the realistic chance of survival for many cardiac arrest victims. Ensuring that these students graduate with robust, practical resuscitation skills is therefore essential for addressing regional health disparities.

Despite the identified knowledge gaps, students demonstrated positive attitudes toward CPR and strong motivation to improve their skills. Confidence levels, however, remained moderate, reinforcing the need for regular hands-on practice to strengthen both competence and self-efficacy [16,46,47,48]. It is important to note that the confidence reported by students reflects their perceived ability rather than objectively demonstrated CPR competence. This distinction is particularly relevant when interpreting their readiness to manage real-life resuscitation scenarios, as perceived confidence may not fully capture the psychomotor skills required to deliver high-quality CPR under emergency conditions.

Students expressed positive attitudes toward CPR and strong motivation to improve their skills. Strengthening structured, hands-on training opportunities, such as simulation-based sessions, may therefore help consolidate competence and confidence among healthcare students [49,50].

Interestingly, no particular hesitation emerged among participants regarding the performance of CPR on individuals of the opposite sex, with most students reporting a high level of confidence in intervening. This contrasts with some international evidence suggesting that gender dynamics may influence willingness to perform specific resuscitation actions, particularly mouth-to-mouth ventilation and chest compressions [51,52]. Proper training has been shown to mitigate these differences, supporting the importance of structured educational programs [52].

Legal aspects are frequently cited in the literature as potential barriers to bystander CPR, with fear of legal repercussions reducing individuals’ willingness to intervene [48,53]. In some contexts, more than half of potential rescuers report legal concerns as a primary reason for hesitating to assist strangers [53]. In our sample, several students expressed uncertainty about the legal implications of providing aid and concern about the possibility of causing unintentional harm. Italian legislation offers protection to individuals who provide emergency assistance in good faith, including healthcare students and professionals acting outside formal work environments. However, awareness of these safeguards remains limited, and training programs rarely address this component [54,55]. Evidence shows that informing learners about existing legal protections increases willingness to perform CPR [54,56], suggesting that integrating this content into educational programs may help reduce hesitation.

For this reason, healthcare education programs should integrate explicit instruction on the legal and ethical foundations of emergency care provision. Students need to understand not only their professional and moral responsibility to assist in life-threatening situations but also the legal protections that support them when they do so. Enhancing this knowledge can meaningfully reduce fear and hesitation, ultimately promoting more timely and effective bystander intervention. Similar surveys conducted in other European and international educational settings have reported comparable gaps in CPR and AED knowledge among healthcare students, despite differences in curricular structures and mandatory training requirements. These findings suggest that limited exposure to standardized resuscitation training is a widespread issue across educational systems, reinforcing the need for more structured and uniform CPR education [17,57]. Students showed a strong interest in receiving further CPR and AED training, indicating that motivation is not a barrier to acquiring these competencies. This finding aligns with international evidence showing that health profession students value resuscitation training and are receptive to structured, simulation-based programs [4,16].

The high level of interest observed suggests that targeted educational interventions would be well received and could strengthen both practical skills and perceived confidence. Enhancing theoretical and practical resuscitation training within health-related degree programs is essential to ensure that future professionals are prepared to respond effectively to cardiac emergencies.

### 4.1. Implications for Practice

The findings of this study have several important implications for health-related university education in Calabria and potentially throughout Italy. First, the results strongly support the integration of mandatory, hands-on BLS-D training within the core curriculum of all healthcare degree programs, ideally beginning in the first academic year and reinforced through periodic refresher sessions. Both the European Resuscitation Council and the American Heart Association recommend annual BLS recertification for healthcare providers, acknowledging that psychomotor skills and theoretical knowledge deteriorate rapidly without regular practice.

Second, curriculum planners should prioritize simulation-based learning, with particular emphasis on the practical components most critical to survival: high-quality chest compressions, early defibrillation, and accurate rhythm recognition. The persistent deficits observed in compression-rate knowledge and AED operation indicate that traditional lecture-based approaches are insufficient. Evidence supports the use of high-fidelity simulation and real-time feedback devices capable of objectively assessing compression depth, rate, and recoil, enabling students to develop both technical proficiency and confidence.

Third, institutions should ensure a clearer and more systematic integration of BLS-D training opportunities within the academic pathway, guaranteeing that all students can access structured and uniformly delivered training. A more coordinated curricular plan, incorporating mandatory and regularly scheduled training sessions, may help address the gaps identified in this study.

Finally, targeted educational strategies are needed to address persistent knowledge deficits, particularly in the recognition of non-shockable rhythms and ventilation techniques. Innovative teaching approaches that incorporate interactive modalities and digital tools have shown promising potential in enhancing knowledge retention and may represent an effective strategy for supporting the learning of these more complex topics.

### 4.2. Strengths and Limitations

This study presents several noteworthy strengths. The large sample size, drawn from multiple healthcare degree programs and spanning different academic years, provides a solid and representative overview of the student population within a major Italian university. The use of assessment tools aligned with current international resuscitation guidelines enhances the validity of the findings and ensures comparability with studies conducted in other countries. Furthermore, the multidimensional evaluation, encompassing theoretical knowledge, rhythm recognition, and procedural understanding, offers a detailed and nuanced picture of student competencies rather than a superficial or fragmented assessment.

Some limitations should also be considered when interpreting the results. The single-center design restricts the generalizability of the findings to other Italian universities, particularly those with different curricular structures or training resources. The cross-sectional nature of the study does not allow for tracking individual students’ progression over time, relying instead on comparisons between cohorts at different academic stages. Additionally, the study focused on theoretical knowledge and self-reported attitudes without incorporating objective assessments of practical skills through mannequins or simulation-based evaluations, which would have provided a more direct measure of actual CPR competence. The questionnaire format may also introduce social desirability bias, potentially leading participants to overestimate their knowledge or confidence. This recruitment approach may have introduced selection bias, potentially leading to an overrepresentation of more motivated or engaged students. As a result, the findings may reflect a best-case scenario regarding CPR/AED knowledge and attitudes, further emphasizing the need to strengthen structured resuscitation training within healthcare curricula. Moreover, because the study relied on self-reported confidence rather than objective evaluations of psychomotor performance, conclusions regarding students’ actual preparedness to perform CPR should be interpreted with caution. In addition, a proportion of participants had prior BLS-D training, which may have influenced their level of knowledge and could have contributed to an overestimation of CPR/AED competencies within the overall sample. Finally, the study did not evaluate long-term knowledge retention among students who had previously completed BLS-D training, leaving open the question of how quickly skills and knowledge deteriorate without reinforcement.

## 5. Conclusions

This study highlights important gaps in CPR and AED knowledge among students at the University of Magna Graecia, with fewer than half having received prior formal BLS-D training and significant deficiencies emerging in key areas such as compression rate, rhythm recognition, and defibrillator use. These findings are particularly relevant given the regional context of high cardiovascular mortality, challenging geography, and limited AED availability. At the same time, the clear learning gradient across academic years and the positive impact of structured training indicate that targeted educational interventions can effectively enhance student competencies. Strengthening mandatory, hands-on BLS-D training within health-related curricula is therefore essential to ensure that future professionals are adequately prepared to respond to cardiac emergencies and contribute to improving survival outcomes in the communities they will serve.

## Figures and Tables

**Table 1:** Attitudes, perceived safety, barriers, and interest in CPR training among healthcare students.

Size	N/Average	%
ETHICAL PERCEPTION		
Moral duty	493	81.6
Depends on the situation	87	14.4
Only if trained	18	3.0
PERCEIVED SAFETY (average scale 1–5)		
Recognizing cardiac arrest	3.31	Low (1–2): 22.7%
Performing CPR	2.83	Low (1–2): 40.6%
Use AED	2.6	Low (1–2): 49.5%
Manage emergency	2.73	Low (1–2): 43.9%
WILLINGNESS TO INTERVENE		
Yes, always	287	47.5
Yes, if trained	267	44.2%
Total available	554	91.7
MAIN BARRIERS		
Lack of training	312	51.7
Fear of making mistakes	178	29.5
Fear of legal consequences	67	11.1
INTEREST IN TRAINING		
Very interested	398	65.9
Interested	167	27.6
Total interested	565	93.5

## Data Availability

The data presented in this study are available on request from the corresponding author. The data are not publicly available due to ethical and privacy restrictions.

## Abbreviations

The following abbreviations are used in this manuscript:

AED	Automated External Defibrillator
AHA	American Heart Association
BLS	Basic Life Support
BLS-D	Basic Life Support and Defibrillation
CPR	Cardiopulmonary Resuscitation
ERC	European Resuscitation Council
KAP	Knowledge–Attitude–Practice
OHCA	Out-of-Hospital Cardiac Arrest
PEA	Pulseless Electrical Activity
